# Identification of Metabolites of Aurantio-Obtusin in Rats Using Ultra-High-Performance Liquid Chromatography-Q-Exactive Orbitrap Mass Spectrometry with Parallel Reaction Monitoring

**DOI:** 10.1155/2021/6630604

**Published:** 2021-04-15

**Authors:** Shi-han Qin, Yuan Xu, Kai-lin Li, Kai-yan Gong, Jie Peng, Si-lin Shi, Fang Yan, Wei Cai

**Affiliations:** ^1^School of Pharmacy, Weifang Medical University, Weifang 261000, China; ^2^School of Pharmaceutical Sciences, Hunan Province Key Laboratory of Antiboby-Based Drug and Intelligent Delivery System, Hunan University of Medicine, Huaihua 418000, China; ^3^Department of TCM Rheumatism, China-Japan Friendship Hospital, Beijing 100029, China

## Abstract

Aurantio-obtusin (AO) is a major anthraquinone compound isolated from *Cassiae Semen* or *Duhaldea nervosa*, which possesses diverse pharmacological effects. Previous studies have shown that it has a good effect on lowering blood lipids and treating various diseases. A few studies have also reported about its metabolites. A rapid and reliable method using ultra-high-performance liquid chromatography-Q-Exactive Orbitrap mass spectrometry and multiple data-processing technologies was established to investigate the metabolites of AO in the plasma and various tissues of rats, including the heart, liver, spleen, lung, kidneys, and brain. Finally, a total of 36 metabolites were identified in the plasma of rats, which could be very beneficial for understanding the effective form of AO metabolites leading to new drug discovery. The result demonstrated that this strategy, especially parallel reaction monitoring, has shown a wide range of applications in the identification of metabolites.

## 1. Introduction

Aurantio-obtusin (AO) is a lipophilic anthraquinone compound, which is isolated from traditional Chinese medicine such as *Cassiae Semen* and *Duhaldea nervosa* [1, 2]. They are both edible and medicinal plants, which have been used for the treatment of hyperlipidemia, hypertensive and rheumatoid arthritis, etc. [3–6]. AO possesses a variety of biological activities, such as antihypertensive activity, antiallergic responses, and anti-inflammatory activity [7, 8]. In recent years, research on AO has been increasing, focusing on the pharmaceutical analysis *in vitro* and pharmacological studies [8, 9]; however, a few *in vivo* investigations have also been done, especially on the metabolites of AO in rats. A total of 21 metabolites of AO were identified in plasma [10, 11]. Therefore, it is of great significance to investigate the metabolism of AO *in vivo*.

Nowadays, ultra-high-performance liquid chromatography coupled with high-resolution mass spectrometry has become one of the essential techniques for the detection and characterization of metabolites, especially for the identification of metabolites in trace amounts or in complex samples [12–15]. Generally, the full scan with data dependence MS^2^ was adopted for the MS^n^ acquiring of the sample. However, the MS^2^ of the metabolites in trace amounts could not be generated in this mode because the MS^2^ will not be triggered if the abundance of the ions in MS^1^ was not reached to the top of 3 [16, 17]. Thus, the metabolites were not completely investigated due to the limitations of the analytical method. In recent years, parallel reaction monitoring (PRM) scanning mode has been developed for achieving the designed ion's MS^2^ data [18–20].

Therefore, a systematic analytical strategy for the metabolites of AO was proposed based on UHPLC Q Exactive Orbitrap with PRM scanning mode. Finally, a total of 36 metabolites were identified in the plasma of rats, which will be very beneficial for understanding the effective form of AO and new drug discovery. The result demonstrated that this strategy, especially parallel reaction monitoring, has shown the ability of a wide range of applications in the identification of metabolites.

## 2. Materials and Methods

### 2.1. Chemicals and Materials

LC-MS grade acetonitrile, methanol, and formic acid (FA) were purchased from Aladdin Industrial Co., Ltd. (Shanghai, China). Deionized water was provided by A.S. Watson Group Ltd. (Hong Kong). All other chemicals used were of analytical grade and obtained from the commercial market. AO standard substance (purity >99.5%) was obtained from Chengdu Pufei De Biotech Co., Ltd. (Chengdu, China).

### 2.2. Animals Experiment

Eight male SD rats (200 ± 20) were provided by Hunan SJA Laboratory Animal Company, China. The rats were housed in a controlled room at standard temperature (24 ± 2°C) and humidity (70 ± 5%) for a week. During this time, free access to food and water was provided to the rats to adapt to the environment. Then, the rats were randomly divided into the drug group and the blank group to assess the plasma and various tissues, including heart, liver, spleen, lung, kidney, and brain. The rats were fasted for 12 h with free access to water prior to the experiment. The animal protocols were approved by the Medicine Ethics Review Committee for Animal Experiments at the Hunan University of Medicine. The animal facilities and protocols complied with the Guide for the Care and Use of Laboratory Animals.

### 2.3. Drug Administration and Collection of Biological Samples

AO was dissolved in 0.5% carboxymethylcellulose sodium (CMC-Na) solution and orally administrated to rats in the drug group at a dose of 200 mg/kg, while 0.5% CMC-Na aqueous solution (2 mL) was administered to rats in the blank group. 0.5 mL of the blood samples was collected from the external jugular vein after the rats were given the drug orally and kept in heparinized tubes for 0.5, 1, 2, and 4 h, and then the plasma was obtained by centrifuging the tubes at 3000 rpm for 15 min at 4°C, respectively. The organs, including heart, liver, spleen, lung, kidney, and brain, were, respectively, harvested from rats in drug and blank groups at 4 h after dosing and washed with cold biological saline. All of the samples were stored at −80°C before further sample pretreatment.

### 2.4. Sample Preparation

In order to detect a maximum number of metabolites of AO, three preparation methods were performed in this study. The first method was processed by solid-phase extraction (SPE). The SPE cartridges were activated and equilibrated by eluting with methanol (5 mL) and deionized water (5 mL) successively. Then, the plasma sample (0.1 mL) was loaded on the SPE column, followed by flushing with deionized water (3 mL) and methanol (3 mL). Afterward, the methanol eluted was collected and dried under the stream of nitrogen at room temperature to gain the residues, which were reconstituted in 0.1 mL of methanol/deionized water (9 : 1) and centrifuged at 12000 rpm for 15 min. The last method used different kinds of organic solvents (methanol or acetonitrile) to precipitate protein. The plasma sample (0.1 mL) was added in a threefold organic solvent and vortexed at 2000 rpm for 1 min. Then, the sample was centrifuged at 12000 rpm for 15 min to obtain the supernatant, which was transferred to a clean tube and dried in an N_2_ stream of nitrogen at room temperature. The residues were reconstituted and centrifuged under the sample condition mentioned above. All the final supernatant was injected into the UHPLC-Q-Exactive Orbitrap for data acquiring.

Each organ, including the heart, liver, spleen, lung, kidney, and brain, was cut into pieces and 0.2 g of mixed samples of each organ was homogenized in 5 volumes of ice-cold saline and centrifuged at 14,000 rpm for 10 min to get the supernatant as tissue samples. A total of 1 mL tissue sample was further processed by the second method described above.

### 2.5. Instruments and Conditions

The chromatographic analysis of all samples was performed with a Thermo Scientific Hypersil GOLD C18 column (100 × 2.1 mm, 1.9 *µ*m) using an Ultimate 3000 UPLC system (Thermo Fisher Scientific, San Jose, CA, USA). 0.1% formic acid aqueous solution (solvent A) and acetonitrile (solvent B) were used as mobile phases with a flow rate of 0.30 mL/min. The flow rate was set at a linear gradient as follows: 0–2 min, 2% B; 2-3 min, 2–25% B; 3–8 min, 25–30% B; 8–12 min, 30–60% B; 12–15 min, 60–80% B; 15–17 min, 80–2% B; 17–20 min, 2% B. The injection volume was 2 *μ*L.

The high-resolution, accurate-mass analysis was performed on Q-Exactive Focus Orbitrap MS (Thermo Electron, Bremen, Germany) with heated electrospray ionization (ESI) source in the negative ions mode. The optimized tune method was set as follows: the flow rate of sheath gas (nitrogen, purity ≥ 99.99%) and auxiliary gas (nitrogen, purity ≥ 99.99%) was set at 30 and 10 arbitrary units, respectively; the temperatures of auxiliary gas heater and capillary were 350 and 320°C, respectively; the voltage of spray was 35 KV; and the S-lens RF level was set at 50. The full MS scan data were acquired at a mass range of m/*z* from 100 to 1000 at a resolving power of 70,000 to screen potential metabolites. The MS^2^ data were obtained at parallel reaction monitoring scanning mode for the identification of metabolites. The collision energy of collision gas (nitrogen, purity ≥ 99.999%) for collision-induced dissociation (CID) was adjusted to 30%.

### 2.6. Data Processing

All raw data were processed by the Thermo Xcalibur software version 4 and the Compound Discover software version 3. The chemical formulas for all parent and fragment ions of the selected peaks were speculated by the accurate mass using a formula predictor by setting the parameters as follows: C[0–40], H[0–90], O[0–30], S[0–3], and N[0–3]. The maximum mass tolerance was set at 5 ppm. Blank biological samples were used as controls for comparison with the analytic samples, and they were all processed under the same conditions.

## 3. Results and Discussion

### 3.1. Analytical Strategy

In this study, all the plasma samples were prepared by the three methods mentioned above to obtain the supernatant, which was injected into UHPLC-Q-Exactive Orbitrap MS to acquire the high-resolution full mass data with the full mass scanning mode. Then, data mining was processed by the Compound Discover workstation using the metabolism workflow template to detect the potential ions based on the biotransformation reactions. Subsequently, the MS^2^ of potential ions was acquired based on the parallel reaction monitoring mode triggered by the potential ions. Finally, the AO metabolites were characterized based on the retention time, accurate full mass, the fragmentation of MS^2^, and bibliography.

### 3.2. Fragmentation Patterns of AO

**Figure 1:**
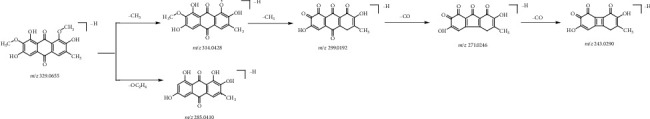
The fragmentation pattern of AO in negative mode.

In order to completely investigate the metabolites of AO, the fragmentation patterns of AO were determined based on the UHPLC-Q-Exactive Orbitrap MS in negative mode. AO showed a deprotonated ion [M-H]^−^ at *m/z* 329.06631 (−1.11 ppm, C_17_H_13_O_7_) and the fragmentation ions at *m/z* 314.0428 (−1.28 ppm, C_16_H_10_O_7_), 299.0192 (−1.76 ppm, C_15_H_7_O_7_), and 285.0410 (1.89 ppm, C_15_H_9_O_6_) by the loss of CH_3_, 2CH_3_, and OC_2_H_4_, respectively, which were selected as the characteristic neutral loss for the metabolite identification. The fragmentation pattern of AO is proposed in Figure 1.

### 3.3. Comparison of the Different Sample Preparation Methods

In this study, three methods were employed to prepare the samples, and then all of them were applied to the UHPLC-Q-Exactive Orbitrap MS under the same condition. According to the retention time, accurate full mass, the fragmentation of MS^2^, and bibliography, a total of 21, 36, and 36 metabolites were screened and detected in the rat plasma using methods 1, 2, and 3, respectively, as shown in Table 1S.

To the best of our knowledge, the above-mentioned methods have been widely used to pretreat a biological sample, holding a great significance in the sample pretreatment. On the basis of the results, the second and the last methods displayed the same results of sample pretreatment, while sample pretreatment by method 1 showed fewer metabolites. After comparison, the second method was chosen as the final preparation method due to the economic solvent.

### 3.4. Identification of AO Metabolites

**Figure 2:**
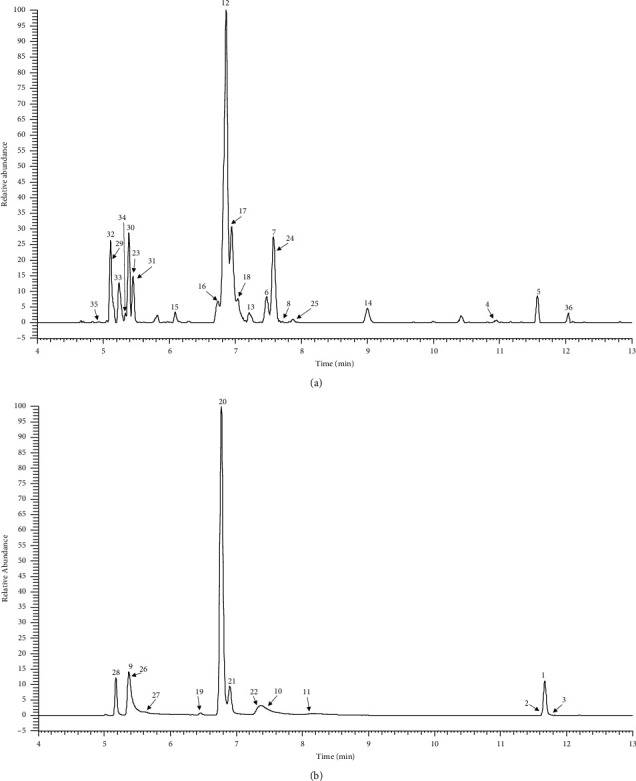
The high-resolution extraction ion chromatography of AO metabolites: (a) *m/z* 299.05611, 315.05102, 345.06159, 475.0882, 491.08311, 521.09258, 651.12029, 667.1152, 697.12577; (b) *m/z* 409.02326, 329.06557, 505.09876, 585.05418, 681.13109.

A total of 36 metabolites (AO included) were tentatively characterized by means of the UHPLC-Q-Exactive Orbitrap MS with the PRM mode. Among them, 22 metabolites were detected for the first time. The high-resolution extraction ion chromatography of these metabolites is shown in Figure 2. The detailed information of these metabolites, including the retention time, the accurate mass, and fragmentation ions, is listed in Table 1.

Metabolite 1 was accurately identified as AO by comparing the retention time, accurate mass, and MS^2^ data with the reference substance. Metabolites 2 and 3 possessed the same MS information, including MS^1^ and MS^2^ and different chromatography behavior with the AO; thus, they were identified as isomers of AO. Metabolites 9–11 showed the deprotonated molecular ions at *m/z* 409.02316 (−0.81 ppm, C_17_H_13_O_10_S), 409.02307 (−1.10 ppm, C_17_H_13_O_10_S), and 409.02322 (−0.04 ppm, C_17_H_13_O_10_S), respectively, with 80 Da greater than that of AO, which suggested the presence of sulfate moiety. In these MS^2^ spectra, fragmentation ions at *m/z* 329.066 (C_17_H_13_O_7_), 314.043 (C_16_H_10_O_7_), and 299.019 (C_15_H_7_O_7_) by the loss of SO_3_, SO_3_ + CH_3_, and SO_3_ + 2CH_3_, respectively, further confirmed the presence of sulfate moiety. Thus, they were assigned to sulfation of AO. Metabolites 19–22 and 26–28 detected the deprotonated molecular ions at *m/z* 505.09866 (−0.21 ppm, C_23_H_21_O_13_), *m/z* 505.09845 (−0.62 ppm, C_23_H_21_O_13_), *m/z* 505.09854 (−0.44 ppm, C_23_H_21_O_13_), *m/z* 505.09860 (−0.32 ppm, C_23_H_21_O_13_), *m/z* 585.05524 (−0.07 ppm, C_23_H_21_O_16_S), *m/z* 585.05524 (−0.07 ppm, C_23_H_21_O_16_S), and *m/z* 681.13062 (−2.30 ppm, C_29_H_29_O_19_) and yielded the same fragmentation with metabolites 9–11, suggesting the same AO moiety. Therefore, metabolites 19–22, 26–27, and 28 were tentatively identified as glucuronidation of AO, glucuronidation and sulfation of AO, and diglucuronidation of AO, respectively.

Metabolites 4 and 5 were eluted at 10.96 and 11.57 min, with the deprotonated molecular ions at *m/z* 299.05530 (−2.71 ppm, C_16_H_11_O_6_) and 299.05603 (−0.27 ppm, C_16_H_11_O_6_), respectively, with 30 Da less than the parent drug. The fragmentation ions at 284.032 (C_16_H_11_O_6_) were generated by the neutral loss of CH_3_. Thus, they were tentatively characterized by demethoxylation of AO. Metabolites 12–13 showed the same quasimolecular ions of *m/z* 475.087 (C_22_H_19_O_12_), which further generated the MS^2^ ions at *m/z* 299.056 (C_16_H_11_O_6_), and 284.032 (C_15_H_8_O_6_) by the loss of glucuronic acid and glucuronic acid + methyl moiety. Therefore, they were identified as glucuronidation and demethoxylation of AO. Besides, metabolites 29–31 were preliminarily characterized by diglucuronidation and demethoxylation of AO.

Metabolites 6–8 were detected at 7.47, 7.57, and 7.75 min and possessed the quasimolecular ions of *m/z* 345.06104 (0.55 ppm, C_17_H_13_O_8_), 345.06094 (0.45 ppm, C_17_H_13_O_8_), and 345.06104 (0.55 ppm, C_17_H_13_O_8_), respectively. They yielded the same MS^2^ ions, including 315.014 (C_15_H_7_O_8_) and 330.037 (C_16_H_7_O_8_) by the neutral loss of 2CH_3_ and CH_3_, respectively, which showed the same fragmentation pattern with AO. Therefore, they were referred to as hydroxylation of AO. Metabolites 23–25 were eluted at 5.44, 7.57, and 7.85 min, respectively, with the deprotonated molecular ions at *m/z* 521.09387 (0.37 ppm, C_23_H_21_O_14_), 521.09332 (−0.69 ppm, C_23_H_21_O_14_), and 521.09241 (−2.43 ppm, C_23_H_21_O_14_), respectively, with 176 Da (C_6_H_8_O_6_, glucuronic acid moiety) more than that of metabolites 6–8, suggesting that they were characterized by glucuronidation of metabolites 6–8. The fragmentation ions at *m/z* 345.0608 (C_17_H_13_O_8_) and 330.0375 (C_16_H_10_O_8_) were detected in the MS^2^ spectrum, which further confirmed that they were characterized by glucuronidation and hydroxylation of AO. Lastly, metabolites 34 and 35 were plausibly characterized by diglucuronidation and hydroxylation of AO.

Metabolite 36, with a retention time of 12.08 min, produced the ions at *m/z* 315.05042 (−1.92 ppm, C_16_H_11_O_7_), with 14 Da less than that of AO. The base peak at *m/z* 300.0273 (−0.84 ppm, C_15_H_8_O_7_) in its MS^2^ spectrum was yielded by the characteristic neutral loss of CH_3_. Therefore, metabolite 36 was characterized by demethylation of AO. Metabolites 15–18 generated the quasimolecular ions of *m/z* 491.08316 (0.09 ppm, C_22_H_19_O_13_), 491.08292 (−0.39 ppm, C_22_H_19_O_13_), 491.08246 (−1.33 ppm, C_22_H_19_O_13_), and 491.08279 (−0.66 ppm, C_22_H_19_O_13_), respectively, with 176 Da greater than that of metabolite 36. The fragmentation ions at m/*z* 315.050 (C_16_H_11_O_7_) and 300.027 (C_15_H_8_O_7_) were yielded by the loss of 176 (C_6_H_8_O_6_) and 191 (C_6_H_8_O_6+_CH_3_), respectively. Therefore, they were tentatively characterized by glucuronidation and demethylation of AO. Likewise, metabolites 32–33 were plausibly characterized by diglucuronidation and demethylation of AO.

### 3.5. Proposed Metabolic Pathways of AO

**Figure 3:**
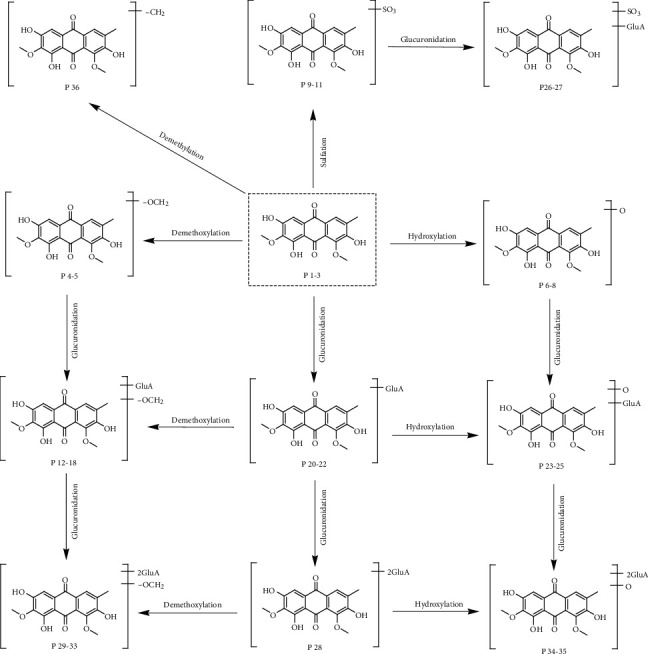
The proposed pathways of AO in the rat plasma.

In this study, the major metabolic pathways of AO in rat plasma are displayed in Figure 3. In general, AO could adopt three kinds of metabolic pathways. The first pathway is Phase I, including demethoxylation (M4-5), hydroxylation (M6-8), and demethylation (M36). The second pathway is Phase II, including sulfation (M9-11), glucuronidation (M19-22), sulfation and glucuronidation (M26-27), and diglucuronidation (M28). The last pathway is the combination of Phases I and II, including glucuronidation and demethoxylation (M12-14), glucuronidation and demethylation (M15-18), glucuronidation and hydroxylation (M23-25), diglucuronidation and demethoxylation (M29-31), diglucuronidation and demethylation (M32-33), and diglucuronidation and hydroxylation (M34-35).

### 3.6. Distribution of AO Metabolites in Rats' Tissues

To the best of our knowledge, the distribution of AO metabolites was investigated for the first time. A total of 16, 15, 10, 15, 16, and 9 metabolites were detected and identified in the heart, liver, spleen, lung, kidney, and heart, respectively (Table 1S). Most of the metabolic reactions, including reactions of Phases I and II, were observed in these organs, which indicated that AO metabolites are widely distributed in all these organs. Metabolites 1, 5, 11, 12, 20, 21, 28, and 36 were distributed in all these organs, suggesting that these metabolites might be the effective form of AO metabolites for exerting pharmacological effects.

## 4. Conclusion

In this study, an effective strategy based on UHPLC-Q-Exactive Orbitrap MS combined with PRM data acquiring was established for the detection and identification of AO metabolites in rats. Finally, a total of 36 metabolites, including phase I and phase II, were characterized in the rat plasma, out of which 22 were reported for the first time. The corresponding reactions, including demethoxylation, hydroxylation, demethylation, sulfation, glucuronidation, and combination reactions, were observed in this study. The study demonstrated that this strategy is useful for the detection of AO metabolites in various biological samples.

## Figures and Tables

**Table 1:** The retention time and mass spectrometric data of AO metabolites.

Peak	*t*_R_	Theoretical mass *m/z*	Experimental mass *m/z*	Error (ppm)	Formula [M-H]^−^	MS/MS fragment	Identification/reactions
1	11.67	329.06557	329.06631	−1.11	C_17_H_13_O_7_	MS^2^[329]:314.0428(100), 299.0192(43), 285.0410(10), 271.0246(3), 243.0290(1)	AO
2	11.49	329.06557	329.06743	2.29	C_17_H_13_O_7_	MS^2^[329]:314.0429(100), 299.0190(35), 285.0412(5)	Isomer of AO
3	11.86	329.06557	329.06589	−2.39	C_17_H_13_O_7_	MS^2^[329]: 299.0197(100), 314.0431(42), 271.0246(39), 285.0408(10)	Isomer of AO
4	10.96	299.05611	299.05530	−2.71	C_16_H_11_O_6_	MS^2^[299]: 284.0320(100)	Demethoxylation of AO
5	11.57	299.05611	299.05603	−0.27	C_16_H_11_O_6_	MS^2^[299]: 284.0323(100)	Demethoxylation of AO
6	7.47	345.06159	345.06104	0.55	C_17_H_13_O_8_	MS^2^[345]: 315.0145(100), 330.0379(30)	Hydroxylation of AO
7	7.57	345.06159	345.06094	0.45	C_17_H_13_O_8_	MS^2^[345]: 315.0146(100), 330.0374(40)	Hydroxylation of AO
8	7.75	345.06159	345.06104	0.55	C_17_H_13_O_8_	MS^2^[345]:315.0143(100)	Hydroxylation of AO
9	5.34	409.02326	409.02316	−0.81	C_17_H_13_O_10_S	MS^2^[409]: 314.0434 (100), 329.0660 (52)	Sulfation of AO
10	7.37	409.02326	409.02307	−1.1	C_17_H_13_O_10_S	MS^2^[409]: 329.0662(100), 314.0430 (97), 299.0197 (6)	Sulfation of AO
11	8.16	409.02326	409.02322	−0.04	C_17_H_13_O_10_S	MS^2^[409]:314.0430 (100), 329.0663 (40), 299.0192 (28)	Sulfation of AO
12	6.86	475.08820	475.08728	−1.94	C_22_H_19_O_12_	MS^2^[475]: 299.0557(100), 284.0323(38)	Glucuronidation and demethoxylation of AO
13	7.21	475.08820	475.08728	−1.94	C_22_H_19_O_12_	MS^2^[475]: 299.0553(100), 284.0324(62)	Glucuronidation and demethoxylation of AO
14	8.99	475.08820	475.08829	0.19	C_22_H_19_O_12_	MS^2^[475]: 299.0553(100), 284.0324(18)	Glucuronidation and demethoxylation of AO
15	6.09	491.08311	491.08316	0.09	C_22_H_19_O_13_	MS^2^[491]: 315.0504(100), 300.0273(38)	Glucuronidation and demethylation of AO
16	6.72	491.08311	491.08292	−0.39	C_22_ H_19_ O_13_	MS^2^[491]: 315.0509(100), 300.0275(98)	Glucuronidation and demethylation of AO
17	6.94	491.08311	491.08246	−1.33	C_22_ H_19_ O_13_	MS^2^[491]: 315.0509(100), 300.0273(92)	Glucuronidation and demethylation of AO
18	7.04	491.08311	491.08279	−0.66	C_22_ H_19_ O_13_	MS^2^[491]: 315.0504(100), 300.0269(48)	Glucuronidation and demethylation of AO	
19	6.46	505.09876	505.09866	−0.21	C_23_H_21_O_13_	MS^2^[505]:314.0426 (100), 329.0660 (44), 299.0191 (8)	Glucuronidation of AO	
20	6.77	505.09876	505.09845	−0.62	C_23_H_21_O_13_	MS^2^[505]:314.0430 (100), 329.0664 (44), 299.0189 (9)	Glucuronidation of AO	
21	6.90	505.09876	505.09854	−0.44	C_23_H_21_O_13_	MS^2^[505]:314.0430(100), 329.0664(60), 299.0191(7)	Glucuronidation of AO	
22	7.30	505.09876	505.09860	−0.32	C_23_H_21_O_13_	MS^2^[505]: 329.0661(100), 314.0426(70)	Glucuronidation of AO	
23	5.44	521.09258	521.09387	0.37	C_23_H_21_O_14_	MS^2^[521]: 345.0608(100), 330.0375(48)	Glucuronidation and hydroxylation of AO	
24	7.57	521.09258	521.09332	−0.69	C_23_H_21_O_14_	MS^2^[521]: 330.0379(100), 345.0611(83), 315.0140 (13)	Glucuronidation and hydroxylation of AO	
25	7.85	521.09258	521.09241	−2.43	C_23_H_21_O_14_	MS^2^[521]: 345.0611(100), 330.0369(80)	Glucuronidation and hydroxylation of AO	
26	5.36	585.05418	585.05524	−0.07	C_23_H_21_O_16_S	MS^2^[585]: 329.0663(100), 314.0431(28), 299.0197(2)	Glucuronidation and sulfation of AO	
27	5.60	585.05418	585.05524	−0.07	C_23_H_21_O_16_S	MS^2^[585]: 329.0663(100), 314.0432(26), 299.0196(1)	Glucuronidation and sulfation of AO	
28	5.17	681.13109	681.13062	−2.30	C_29_H_29_O_19_	MS^2^[681]: 329.0662(100), 314.0429(38), 299.0197(2)	Diglucuronidation of AO	
29	5.15	651.12029	651.12103	1.14	C_28_H_27_O_18_	MS^2^[651]:284.0322(100), 299.0557(85)	Diglucuronidation and demethoxylation of AO	
30	5.39	651.12029	651.12457	6.58	C_28_H_27_O_18_	MS^2^[651]:284.0325(100), 299.0564(85)	Diglucuronidation and demethoxylation of AO	
31	5.45	651.12029	651.12079	0.77	C_28_H_27_O_18_	MS^2^[651]:299.0569(100)	Diglucuronidation and demethoxylation of AO	
32	5.10	667.11520	667.11713	2.89	C_28_H_27_O_19_	MS^2^[667]:300.0278(100), 315.0514(87)	Diglucuronidation and demethylation of AO	
33	5.22	667.11520	667.11749	3.43	C_28_H_27_O_19_	MS^2^[667]:300.0277(100), 187.0064(97), 315.0515(57)	Diglucuronidation and demethylation of AO	
34	5.33	697.12577	697.12738	2.31	C_29_H_29_O_20_	MS^2^[697]: 345.0613(100), 521.0936(10)	Diglucuronidation and hydroxylation of AO	
35	4.96	697.12577	697.12847	3.88	C_29_H_29_O_20_	MS^2^[697]: 345.0610 (100)	Diglucuronidation and hydroxylation of AO	
36	12.08	315.05102	315.05042	−1.92	C_16_H_11_O_7_	MS^2^[315]: 300.0273 (100)	Demethylation of AO	

## Data Availability

The data used to support the finding of this study are available from the corresponding author upon request.

## Supplementary Materials

Supplementary Materials Table 1S: the distribution of AO metabolites in different samples and different methods.

## References

[1] Yang X., Liu L.-Z., Chen Z.-Z. (2018). Five anthraquinone compounds in Cassia seed were detected by high performance capillary electrophoresis with UV detection. *Chinese Journal of Analysis Laboratory*.

[2] Xiao S.-L., Guan L.-J., Jiang R.-F. (2020). The metabolism and pharmacokinetic of rhein and aurantio-obtusin. *Current Drug Metabolism*.

[3] Xie W., Zhao Y., Du L. (2012). Emerging approaches of traditional Chinese medicine formulas for the treatment of hyperlipidemia. *Journal of Ethnopharmacology*.

[4] Guo M., Liu Y., Gao Z.-Y. (2014). Chinese herbal medicine on dyslipidemia: progress and perspective. *Evid-Based Complementary and Alternative Medicine*.

[5] Xiao C.-W. (1997). Textual research of dong medicine and a famous doctor for treating traumatic injury. *Journal of Medicine & Pharmacy of Chinese Minorities*.

[6] Long S. (2004). Clinical experience of “maoshoucai” for treating traumatic injury. *Journal of Medicine & Pharmacy of Chinese Minorities*.

[7] Kim M., Lim S. J., Lee H.-J., Nho C. W. (2015). Cassia tora seed extract and its active compound aurantio-obtusin inhibit allergic responses in IgE-mediated mast cells and anaphylactic models. *Journal of Agricultural and Food Chemistry*.

[8] Hou J., Gu Y., Zhao S. (2018). Anti-inflammatory effects of aurantio-obtusin from seed of Cassia obtusifolia L. Through modulation of the NF-*κ*B pathway. *Molecules*.

[9] Xu L., Chan C.-O., Lau C.-C., Yu Z., Mok D. K. W., Chen S. (2012). Simultaneous determination of eight anthraquinones in semen Cassiae by HPLC-DAD. *Phytochemical Analysis*.

[10] Xu L.-L. (2017). Effect of semen cassiae on CY450 enzymes and related metabolomics in rat liver.

[11] Xu L.-L., Zhang Z., Hao F.-R. (2021). A comparative study of aurantio-obtusin metabolism in normal and liver-injured rats by ultra performance liquid chromatography quadrupole time-of-flight mass spectrometry. *Journal of Pharmaceutical and Biomedical Analysis*.

[12] Zhang J.-Y., Wang F., Cai W. (2015). Identification of metabolites of gardenin A in rats by combination of high-performance liquid chromatography with linear ion trap-Orbitrap mass spectrometer based on multiple data processing techniques. *Biomedical Chromatography: BMC*.

[13] Shang Z., Wang F., Dai S., Lu J., Wu X., Zhang J. (2017). Profiling and identification of (−)-epicatechin metabolites in rats using ultra-high performance liquid chromatography coupled with linear trap-Orbitrap mass spectrometer. *Drug Testing and Analysis*.

[14] Liang Y., Zhao W., Wang C., Wang Z., Wang Z., Zhang J. (2018). A comprehensive screening and identification of genistin metabolites in rats based on multiple metabolite templates combined with UHPLC-HRMS analysis. *Molecules*.

[15] Shao W.-J., Shang Z.-P., Li Q.-Q. (2018). Rapid screening and identification of daidzein metabolites in rats based on UHPLC-LTQ-orbitrap mass spectrometry coupled with data-mining technologies. *Molecules*.

[16] Liu L., Zhang J., Zheng B. (2018). Rapid characterization of chlorogenic acids in Duhaldea nervosa based on ultra-high-performance liquid chromatography-linear trap quadropole-Orbitrap-mass spectrometry and mass spectral trees similarity filter technique. *Journal of Separation Science*.

[17] Xu R. N., Fan L., Rieser M. J., El-Shourbagy T. A. (2007). Recent advances in high-throughput quantitative bioanalysis by LC-MS/MS. *Journal of Pharmaceutical and Biomedical Analysis*.

[18] Zhang S.-X., Zhu W.-X., Liu S.-N., Wang X.-J., Zhang S.-J. (2020). Detection of walnut, almond and peanut soybean source components in walnut sauce based on HPLC-Q-Exactive PRM technology. *Food Science and Technology*.

[19] Gong K., Yang Y., Li K., Zhu L., Zhi X., Cai W. (2020). Identification of the metabolites of isochlorogenic acid A in rats by UHPLC-Q-Exactive Orbitrap MS. *Pharmaceutical Biology*.

[20] Cai W., Li K.-L., Xiong P. (2020). A systematic strategy for rapid identification of chlorogenic acids derivatives in *Duhaldea nervosa* using UHPLC-Q-Exactive Orbitrap mass spectrometry. *Arabian Journal of Chemistry*.

