# Supplementary material for: Identification of Metabolites of Aurantio-Obtusin in Rats Using Ultra-High-Performance Liquid Chromatography-Q-Exactive Orbitrap Mass Spectrometry with Parallel Reaction Monitoring
Source: J Anal Methods Chem. 2021 Apr 15;2021:6630604. doi: 10.1155/2021/6630604 (PMC8062173; doi:10.1155/2021/6630604)
Supplement: Supplementary Materials — Table 1S: the distribution of AO metabolites in different samples and different methods. [file 6630604.f1.docx]

Table 1S: The distribution of AO metabolites in different sample and different method

| Peak | t_R_ | Theoretical Mass *m/z* | Formula [M-H]^-^ | Identification/Reactions | T0.5 | T1 | T2 | T4 | Heart | liver | spleen | lung | kidney | brain | 1 | 2 | 3 |
| --- | --- | --- | --- | --- | --- | --- | --- | --- | --- | --- | --- | --- | --- | --- | --- | --- | --- |
| 1 | 11.67 | 329.06557 | C_17_H_13_O_7_ | AO | + | + | + | + | + | + | + | + | + | + | + | + | + |
| 2 | 11.49 | 329.06557 | C_17_H_13_O_7_ | Isomer of AO | + | + | + | + | - | - | - | - | - | - | + | + | + |
| 3 | 11.86 | 329.06557 | C_17_H_13_O_7_ | Isomer of AO | - | + | + | + | - | - | - | - | - | - | + | + | - |
| 4 | 10.96 | 299.05611 | C_16_H_11_O_6_ | Demethoxylation of AO | - | + | + | + | - | - | - | - | - | - | + | + | + |
| 5 | 11.57 | 299.05611 | C_16_H_11_O_6_ | Demethoxylation of AO | + | + | + | + | + | + | + | + | + | + | + | + | - |
| 6 | 7.47 | 345.06159 | C_17_ H_13_ O_8_ | Hydroxylation of AO | + | + | + | + | - | - | - | - | - | - | + | + | + |
| 7 | 7.57 | 345.06159 | C_17_H_13_O_8_ | Hydroxylation of AO | - | + | + | + | - | - | - | - | - | - | + | + | + |
| 8 | 7.75 | 345.06159 | C_17_H_13_O_8_ | Hydroxylation of AO | - | - | - | + | - | - | - | - | - | - | + | + | + |
| 9 | 5.34 | 409.02326 | C_17_H_13_O_10_S | Sulfation of AO | - | - | - | + | - | - | - | - | - | - | + | + | + |
| 10 | 7.37 | 409.02326 | C_17_H_13_O_10_S | Sulfation of AO | + | + | + | + | - | - | - | - | - | - | + | + | + |
| 11 | 8.16 | 409.02326 | C_17_H_13_O_10_S | Sulfation of AO | - | + | + | + | + | + | + | + | + | + | + | + | + |
| 12 | 6.86 | 475.08820 | C_22_H_19_O_12_ | Glucuronidation and Demethoxylation of AO | + | + | + | + | + | + | + | + | + | + | + | + | + |
| 13 | 7.21 | 475.08820 | C_22_H_19_O_12_ | Glucuronidation and Demethoxylation of AO | + | + | + | + | - | - | - | - | - | - | + | + | - |
| 14 | 8.99 | 475.08820 | C_22_H_19_O_12_ | Glucuronidation and Demethoxylation of AO | + | + | + | + | + | - | - | - | - | - | + | + | - |
| 15 | 6.09 | 491.08311 | C_22_H_19_O_13_ | Glucuronidation and Demethylation of AO | - | + | + | + | - | - | - | - | - | - | + | + | - |
| 16 | 6.72 | 491.08311 | C_22_ H_19_ O_13_ | Glucuronidation and Demethylation of AO | + | + | + | + | + | + | - | + | + | - | + | + | + |
| 17 | 6.94 | 491.08311 | C_22_ H_19_ O_13_ | Glucuronidation and Demethylation of AO | + | + | + | + | + | + | + | + | + | + | + | + | + |
| 18 | 7.04 | 491.08311 | C_22_ H_19_ O_13_ | Glucuronidation and Demethylation of AO | - | + | + | + | + | - | - | + | + | - | + | + | - |
| 19 | 6.46 | 505.09876 | C_23_H_21_O_13_ | Glucuronidation of AO | + | + | + | + | + | + | + | + | + | - | + | + | + |
| 20 | 6.77 | 505.09876 | C_23_H_21_O_13_ | Glucuronidation of AO | + | + | + | + | + | + | + | + | + | + | + | + | + |
| 21 | 6.90 | 505.09876 | C_23_H_21_O_13_ | Glucuronidation of AO | + | + | + | + | + | + | + | + | + | + | + | + | + |
| 22 | 7.30 | 505.09876 | C_23_H_21_O_13_ | Glucuronidation of AO | + | + | + | + | + | + | - | + | + | - | + | + | + |
| 23 | 5.44 | 521.09258 | C_23_H_21_O_14_ | Glucuronidation and Hydroxylation AO | + | + | + | + | + | + | - | + | + | - | + | + | - |
| 24 | 7.57 | 521.09258 | C_23_H_21_O_14_ | Glucuronidation and Hydroxylation of AO | + | + | + | + | - | - | - | - | - | - | + | + | - |
| 25 | 7.85 | 521.09258 | C_23_H_21_O_14_ | Glucuronidation and Hydroxylation of AO | - | - | - | + | - | - | - | - | - | - | + | + | - |
| 26 | 5.36 | 585.05418 | C_23_H_21_O_16_S | Glucuronidation and Sulfation of AO | + | + | + | + | - | - | - | - | - | - | + | + | + |
| 27 | 5.60 | 585.05418 | C_23_H_21_O_16_S | Glucuronidation and Sulfation of AO | + | + | + | + | - | - | - | - | - | - | + | + | + |
| 28 | 5.17 | 681.13109 | C_29_H_29_O_19_ | Diglucuronidation of AO | + | + | + | + | + | + | + | + | + | + | + | + | + |
| 29 | 5.15 | 651.12029 | C_28_H_27_O_18_ | Diglucuronidation and Demethoxylation of AO | + | + | + | + | - | + | - | - | + | - | + | + | - |
| 30 | 5.39 | 651.12029 | C_28_H_27_O_18_ | Diglucuronidation and Demethoxylation of AO | + | + | + | + | + | + | - | + | + | - | + | + | - |
| 31 | 5.45 | 651.12029 | C_28_H_27_O_18_ | Diglucuronidation and Demethoxylation of AO | - | + | + | + | - | - | - | - | - | - | + | + | - |
| 32 | 5.10 | 667.11520 | C_28_H_27_O_19_ | Diglucuronidation and demethylation of AO | + | + | + | + | - | - | - | - | - | - | + | + | + |
| 33 | 5.22 | 667.11520 | C_28_H_27_O_19_ | Diglucuronidation and demethylation of AO | + | + | + | + | - | - | - | - | - | - | + | + | - |
| 34 | 5.33 | 697.12577 | C_29_H_29_O_20_ | Diglucuronidation and Hydroxylation of AO | - | - | - | + | - | - | - | - | - | - | + | + | - |
| 35 | 4.96 | 697.12577 | C_29_H_29_O_20_ | Diglucuronidation and Hydroxylation of AO | - | - | - | + | - | - | - | - | - | - | + | + | - |
| 36 | 12.08 | 315.05102 | C_16_H_11_O_7_ | Demethylation of AO | - | - | - | + | + | + | + | + | + | + | + | + | + |

T0.5: 0.5h after drug administration; T1: 1h after drug administration; T2: 2h after drug administration; T4: 4h after drug administration;

1: method 1; 2: method 2; 3: method 3.
